# Stochastic three-term conjugate gradient: a third-order curvature approximation correction algorithmic framework for machine learning

**DOI:** 10.1038/s41598-026-58525-8

**Published:** 2026-06-22

**Authors:** Jiazhen Liu, Gonglin Yuan, Zhongyu Mo

**Affiliations:** 1 Postdoctoral Research Workstation, Guangxi Rural Commercial United Bank, No. 148 Minzu Ave., Nanning, 530022 P. R. China; 2 School of Mathematics, Center for Applied Mathematics of Guangxi, Guangxi University, Nanning, 530004 Guangxi P. R. China

**Keywords:** Stochastic optimization, Three-term conjugate gradient method, Third-order curvature approximation, Variance reduction, Nonconvex optimization, Engineering, Mathematics and computing, Physics

## Abstract

Classical conjugate gradient methods rely solely on first-order information, which limits their ability to capture curvature information in nonconvex stochastic optimization. To address this limitation, this paper proposes a stochastic three-term conjugate gradient algorithm incorporating third-order curvature approximation (TASCG). By integrating third-order tensor information into the search direction, the proposed algorithm enhances its ability to capture the local geometry of nonconvex loss landscapes, while satisfying both the sufficient descent property and boundedness conditions without additional assumptions. Under standard assumptions of gradient Lipschitz continuity and bounded variance, we rigorously establish global convergence of the TASCG algorithm and derive a stochastic first-order oracle complexity bound of $\mathcal {O}(\epsilon ^{-2})$, which matches the optimal complexity of classical stochastic gradient methods. To further improve gradient estimation accuracy, we incorporate variance reduction techniques into the TASCG framework, resulting in the variant TASCG-VR. Numerical experiments on nonconvex SVM and empirical risk minimization problems demonstrate that the proposed algorithms significantly outperform standard SGD and SVRG in terms of convergence speed and final solution accuracy, while being more robust to step-size selection. This work provides a novel theoretical perspective and algorithmic framework for integrating higher-order geometric information into stochastic conjugate gradient methods.

## Introduction

As a cornerstone of modern optimization theory, nonconvex stochastic optimization concerns the minimization of nonconvex objective functions under stochastic perturbations. It plays a vital role across a broad range of engineering disciplines by providing both rigorous theoretical foundations and computational tools for navigating complex optimization landscapes. The problem is canonically formulated as:

1 $$\begin{aligned} \min _{w \in \Re ^n} \ \phi (w) = \mathbb {E}_{\zeta \sim P}[\phi (w;\zeta )], \end{aligned}$$

where $\phi : \Re ^n \times \Re ^m \rightarrow \Re$ denotes a smooth, nonconvex function, and $\phi (w): \Re ^n \rightarrow \Re$ is continuously differentiable. Here, $\mathbb {E}[\cdot ]$ represents the expectation with respect to the random variable *ζ* drawn from distribution *P*. Nonconvex stochastic optimization thus serves as a critical bridge between abstract optimization theory and practical engineering applications.

In machine learning, the Empirical Risk Minimization (ERM) framework forms the foundation for a wide range of predictive tasks, including image recognition, natural language processing, and financial risk modeling, as well as the training of deep neural networks, highlighting its theoretical importance and practical relevance^1–3^. This framework aims to learn predictive models by approximating the expected risk using empirical data, while mitigating overfitting by minimizing a regularized empirical loss function. Formally, the ERM problem can be formulated as:

2 $$\begin{aligned} \min _{w \in \Re ^d} \ \phi (w) = \frac{1}{n}\sum _{i=1}^n \phi _i(w) + \frac{\lambda }{2} \Vert w\Vert ^2, \end{aligned}$$

where $w \in \Re ^d$ denotes the model parameter vector, $\phi _i(w): \Re ^d \rightarrow \Re$ represents the loss associated with the *i*-th sample-typically nonconvex, as in the cross-entropy loss widely used in deep neural networks-and $\lambda \Vert w\Vert ^2$ is an $\ell _2$ regularization term with coefficient *λ > 0*. Notably, contemporary large-scale datasets are characterized by extremely large sample sizes *n*.

In balancing computational efficiency with convergence guarantees, methods for stochastic optimization have evolved along two main directions. The first direction consists of first order methods, most notably stochastic gradient descent (SGD) and its variants, including momentum based methods, adaptive methods, and variance-reduction techniques, which are widely used due to their low per-iteration cost, typically $\mathcal {O}(d)$ for problems of dimension *d*. The second direction includes stochastic conjugate gradient and stochastic quasi-Newton methods, which exploit curvature information via conjugate search directions or gradient differences, often achieving faster convergence in practice while maintaining relatively low per-iteration complexity using only first-order oracle information^4^.

Stochastic Gradient Descent, introduced in the seminal work of Robbins and Monro^5^ in 1951, follows the iterative update:

$$\begin{aligned} w_{k+1} = w_k - \eta _k \nabla \phi _{i_k}(w_k), \end{aligned}$$

where $\eta _k > 0$ is the step size and $i_k$ is a randomly sampled data index. Despite its simplicity and low computational cost, which make it well suited for large-scale nonconvex problems, SGD suffers from sensitivity to step-size selection, oscillatory convergence behavior, and relatively slow asymptotic convergence. To address these limitations, variance reduction techniques have been developed to improve the performance of stochastic gradient methods. The Stochastic Variance Reduced Gradient (SVRG) method, proposed by Johnson and Zhang^6^ in 2013, reduces gradient variance through the periodic computation of a full-gradient reference. Its update rule is given by:

$$\begin{aligned} v_k = \nabla \phi _{i_k}(w_k) - \nabla \phi _{i_k}(\tilde{w}) + \nabla \phi (\tilde{w}), \end{aligned}$$

where $\tilde{w}$ is a periodically updated reference point and $\nabla \phi (\tilde{w})$ is the full gradient evaluated at that point. The related SAGA algorithm, introduced by Defazio et al.^7^, adopts a table-based approach to variance reduction. Further studies by Reddi et al.^8^ analyze variance-reduced methods in asynchronous parallel settings, while Allen-Zhu and Hazan^9^ provide theoretical guarantees for accelerated convergence in nonconvex optimization. Although these methods improve convergence stability by reducing stochastic noise, they remain fundamentally limited by their reliance on first-order information. Consequently, they do not fully capture local curvature properties of nonconvex objectives, which restricts their performance on more complex optimization landscapes^4,10^.

Conjugate gradient methods constitute an efficient class of algorithms for unconstrained optimization. The linear conjugate gradient method, designed for positive-definite quadratic objectives, was first introduced by Hestenes and Stiefel^11^ in 1952. This framework was subsequently extended to general nonlinear optimization by Fletcher and Reeves^12^, as well as Polak and Ribière^13^. The search direction in this class of methods is defined by the recursion:

$$\begin{aligned} d_k = -g_k + \beta _k d_{k-1}, \quad d_0 = -g_0, \end{aligned}$$

where $g_k = \nabla \phi (w_k)$ denotes the gradient at iteration *k*, and $\beta _k$ is a scalar parameter that ensures conjugacy. Common choices for this parameter include the Fletcher–Reeves (FR) formula, $\beta _k^{\textrm{FR}} = \Vert g_k\Vert ^2 / \Vert g_{k-1}\Vert ^2$, and the Polak–Ribière–Polyak (PRP) formula, $\beta _k^{\textrm{PRP}} = g_k^T y_{k-1} / \Vert g_{k-1}\Vert ^2$, where $y_{k-1} = g_k - g_{k-1}$ denotes the difference between successive gradients.

In recent years, an increasing number of studies have focused on combining the direction update framework of conjugate gradient methods with stochastic optimization methods. This line of work has led to the development of stochastic conjugate gradient algorithms, which aim to combine the low per-iteration cost of stochastic approximation with the curvature information implicitly captured by classical conjugate gradient methods. He and Du^14^ introduced the ASCG algorithm, which constitutes the first systematic integration of SVRG-style variance reduction with step-size acceleration factors within a stochastic conjugate gradient scaffold. In the context of strongly convex empirical risk minimization, this work rigorously establishes a linear convergence rate in expectation for the objective gap, addressing a previously unresolved aspect of the theoretical analysis of stochastic conjugate gradient methods. Kou et al.^15^ subsequently advanced a Mini-Variance mini-batch stochastic conjugate gradient algorithm. By constructing a class of unbiased stochastic gradient estimators and discerning the specific estimator that attains the minimal variance, they demonstrated that both variants of their proposed method exhibit linear convergence rates under the canonical assumptions of strong convexity and smoothness. Further extending this line of research, Yimer et al.^16^ proposed a Stochastic Spectral Conjugate Gradient algorithm, which amalgamates variance reduction techniques with spectral conjugate gradient formulations. This approach harmonizes the respective virtues of the FR and PRP parameterizations and furnishes a proof of linear convergence predicated upon smooth, strongly convex objective functions. Moreover, complementary contributions to this evolving landscape encompass a Steffensen-based stochastic conjugate gradient framework advanced by Yang^17^, an investigation into variance reduction techniques for three-term stochastic conjugate gradient methods by Ouyang et al.^18^, and the application of inertial extrapolation coupled with stochastic recursive gradients to nonconvex stochastic optimization problems by Mo et al.^3^. Lastly, Yuan et al.^2^ have provided a novel formulation of stochastic conjugate gradient algorithms derived from the analytical perspective of stochastic differential equations.

Although these algorithms provide new approaches to solving nonconvex stochastic optimization problems, several significant limitations remain. First, the construction of conjugate parameters predominantly adheres to the conventional second-order secant equation, $\nabla ^2 \phi (w_{k}) s_{k-1} \approx y_{k-1}$. The approximation error in this formulation scales as $O(\Vert s_{k-1}\Vert ^2)$, which limits the information it captures to second-order curvature properties. As a consequence, this framework proves inadequate for faithfully delineating the intricate local geometric topography characteristic of nonconvex objective functions. This deficiency engenders substantial bias in parameter estimation and renders the search direction susceptible to entrapment within suboptimal local basins of attraction^16,19^. Second, the preponderance of extant algorithms necessitates the imposition of auxiliary constraints-such as step-size restrictions or the incorporation of preconditioning matrices-as a requisite condition for ensuring the sufficient descent property, namely $g_k^T d_k \le -c \Vert g_k\Vert ^2$ (for some *c > 0*). This reliance on external modifications limits the generality and applicability of such methods, and reduces their adaptability to the varying demands of nonconvex stochastic optimization problems^18,20,21^. Third, within large-scale nonconvex regimes, a persistent tension obtains between the celerity of algorithmic convergence and the stability of the iterative trajectory. Although certain methods employ mini-batch processing to reduce computational cost, the effectiveness of variance reduction is often limited, which reduces the accuracy of the final solution^4,22,23^.

The secant equation provides the theoretical foundation for defining conjugate parameters in conjugate gradient methods. The accuracy of this approximation directly influences both the quality of the resulting search direction and the asymptotic convergence behavior of the algorithm^2,20^. The standard secant equation, derived from second-order information, is given by:

$$\begin{aligned} B_k s_{k-1} = y_{k-1},\end{aligned}$$

where $s_{k-1} = w_k - w_{k-1}$ denotes the step displacement, $y_{k-1} = \nabla \phi (w_k) - \nabla \phi (w_{k-1})$ represents the gradient difference, and $B_k$ approximates the Hessian matrix $\nabla ^2 \phi (w_k)$ . However, this formulation captures only second-order curvature information and does not account for higher-order characteristics inherent in nonconvex objectives. This limitation has been identified as a key factor restricting performance improvements in stochastic conjugate gradient methods^17^.

To address this issue, Nesterov and Polyak^24^ proposed an optimization framework based on third-order tensor approximations. The central idea is to employ a third-order Taylor expansion of the objective function:

$$\begin{aligned} \phi (w_k + d) \approx \phi (w_k) + \nabla \phi (w_k)^T d + \frac{1}{2} d^T \nabla ^2 \phi (w_k) d + \frac{1}{6} \mathcal {T}_k[d,d,d],\end{aligned}$$

where $\mathcal {T}_k$ denotes a third-order tensor. By incorporating higher-order derivative information, this approach improves upon the limitations of second-order approximations and provides a new perspective for enhancing the estimation of conjugate parameters. Subsequently, Birgin et al.^25^ integrated third-order tensor techniques into a quasi-Newton framework, further demonstrating the potential of higher-order information for navigating complex nonconvex optimization landscapes.

In the empirical analysis, with a large number of underlying bonds, time-varying term structure dynamics, and strict risk management requirements, portfolio optimization in the interest rate bond market poses substantial challenges to conventional methods. Standard mean-variance models and basic stochastic gradient algorithms often exhibit unstable convergence and high sensitivity to market noise, especially in high-dimensional allocation settings. To address the aforementioned challenges, this paper combines the enhanced curvature representation provided by third-order tensor information with the robust performance of three-term conjugate gradient methods under inexact line search conditions. Focusing on nonconvex stochastic optimization, we propose a Stochastic Three-term Conjugate Gradient algorithm based on third-order curvature approximation (TASCG). This algorithm provides potentials for improves the stability and accuracy of fixed-income portfolio optimization under dynamic interest rate conditions. The main contributions of this work are summarized as follows:

- By introducing a novel search direction that incorporates third-order tensor information, we develop a stochastic three-term conjugate gradient algorithm based on third-order curvature approximation. The proposed search direction satisfies both the sufficient descent condition and boundedness of the search direction without imposing additional assumptions, which improves the generality and numerical stability of the method.
- In nonconvex stochastic optimization, we establish global convergence guarantees for the proposed algorithm. The analysis combines descent lemmas in expectation with martingale convergence techniques and provides an iteration complexity analysis, which strengthens the theoretical foundation of the method.
- Variance reduction techniques are integrated into the proposed stochastic conjugate gradient algorithm to reduce the variance of stochastic gradient estimates while maintaining computational efficiency. Extensive numerical experiments on standard benchmark datasets demonstrate the effectiveness and practical applicability of the method in nonconvex machine learning tasks, highlighting the advantages of incorporating third-order curvature information.

The remainder of this paper is organized as follows. “Algorithm and motivation” describes the design rationale and underlying motivation of the proposed algorithm. “Convergence analysis and iteration complexity of the algorithm” presents a systematic analysis of its convergence properties and computational complexity. “Numerical experiments” provides a comprehensive numerical evaluation of the algorithm’s performance. Finally, “Conclusion” concludes the paper and outlines directions for future research.

## Algorithm and motivation

This section commences with an exposition of the conceptual motivation underpinning the proposed algorithmic framework, followed by a detailed delineation of the Stochastic Three-term Conjugate Gradient Method predicated on Third-order Curvature Approximation (TASCG).

### Motivation and algorithmic construction

The classical conjugate gradient method, when implemented with an exact line search, satisfies the orthogonality condition $g_k^T d_{k-1} = 0$. This property plays a fundamental role. First, it guarantees finite-step convergence when applied to convex quadratic objective functions. Second, it enables a simplified two-term recursion for the search direction, $d_k = -g_k + \beta _k d_{k-1}$, which underpins the computational efficiency of conjugate gradient methods. In practice, however, exact line search is often infeasible, and the orthogonality condition no longer holds. When $g_k^T d_{k-1} \ne 0$, the influence of the previous search direction persists, disrupting conjugacy between successive directions. This loss of conjugacy can significantly degrade convergence performance, leading to numerical instability or slower convergence.

In response to this challenge, substantial research efforts have been devoted to developing variants of the conjugate gradient method that retain global convergence under inexact line search conditions. Notable directions include the design of three-term extensions that explicitly enforce descent^26^, the incorporation of spectral scaling to accelerate convergence^27^, and the derivation of revised parameter formulations for $\beta _k$^28,29^, with recent work also extending these ideas to statistical regression frameworks^30^.

Among these developments, the three-term conjugate gradient formulation stands out as a particularly direct mechanism for mitigating the loss of conjugacy. The core idea is to introduce a simple yet effective correction term that explicitly compensates for the non-orthogonality induced by inexact line searches. Specifically, while retaining the curvature information encoded in the conjugate parameter $\beta _k$, the method augments the search direction with an orthogonality correction term:

$$\begin{aligned} d_k = -g_k + \beta _k d_{k-1} - \frac{g_k^T d_{k-1}}{\Vert d_{k-1}\Vert ^2} d_{k-1}. \end{aligned}$$

This additional term subtracts the projection of the current gradient $g_k$ onto the previous direction $d_{k-1}$, which restores approximate conjugacy under inexact line search. The descent property of the modified direction can be verified by examining:

$$\begin{aligned} g_k^T \left( -g_k - \frac{g_k^T d_{k-1}}{\Vert d_{k-1}\Vert ^2} d_{k-1} \right) = -\Vert g_k\Vert ^2 - \frac{(g_k^T d_{k-1})^2}{\Vert d_{k-1}\Vert ^2} \le -\Vert g_k\Vert ^2.\end{aligned}$$

Combined with the conjugate term $\beta _k d_{k-1}$, and assuming that $\beta _k$ is properly chosen to control the behavior of $g_k^T d_{k-1}$, this result shows that the correction term preserves-and can even strengthen-the descent property of the search direction. Despite these advantages, both the conjugate parameter $\beta _k$ and the orthogonality correction rely solely on first-order gradient information and therefore do not fully capture the curvature of the objective function. In highly nonlinear or ill-conditioned problems, this limitation can restrict the achievable convergence rate.

To address this limitation, this paper proposes a novel three-term conjugate gradient method enhanced by a third-order tensor correction. Building on the classical three-term framework, the search direction is defined as:

3 $$\begin{aligned} d_k = -g_k + \beta _k^{\textrm{NE}+} d_{k-1} - \rho \frac{g_k^T d_{k-1}}{\delta _k} y_{kt}, \end{aligned}$$

where $\rho \in (0,1)$ is a scalar parameter, and the conjugate coefficient $\beta _k^{\textrm{NE}+}$ is given by:

$$\begin{aligned} \beta _k^{\textrm{NE}+} = \max \{ \frac{g_k^T y_{kt}}{\delta _k}, 0 \} - \frac{t g_k^T s_k}{\delta _k}, \end{aligned}$$

with normalization factor

$$\begin{aligned} \delta _k = \max \{\Vert d_{k-1}\Vert \Vert y_{kt}\Vert , \Vert d_{k-1}\Vert \Vert s_k\Vert \}.\end{aligned}$$

Unlike standard approaches, the vector $y_{kt}$ is not simply the gradient difference $y_k = \nabla \phi _{k+1} - \nabla \phi _k$. Instead, it is refined by incorporating third-order tensor information, yielding:

4 $$\begin{aligned} y_{kt} = y_k + \frac{6(\phi _k - \phi _{k+1}) + 3(\nabla \phi _k + \nabla \phi _{k+1})^T s_k}{5\Vert s_k\Vert ^2} s_k. \end{aligned}$$

This construction is derived from a third-order Taylor expansion of the objective function *ϕ* around $w_{k+1}$:

$$\begin{aligned} \phi (w_{k+1} + d) = \phi _{k+1} + \nabla \phi _{k+1}^T d + \frac{1}{2} d^T \nabla ^2 \phi _{k+1} d + \frac{1}{6} d^T (H_{k+1} d) d + O(\Vert d\Vert ^4),\end{aligned}$$

where $H_{k+1}$ denotes the third-order derivative tensor. To avoid the explicit evaluation of this tensor, we adopt the approximation strategy introduced by Chow et al.^31^, in which the third-order term is rendered tractable through an expression involving only lower-order quantities. Invoking the interpolation conditions $\nabla \phi _k = \nabla \phi _B(w_{k+1} - s_k)$ and $\phi _k = \phi _B(w_{k+1} - s_k)$, where $\phi _B$ denotes the objective function evaluated over a mini-batch *B*, together with algebraic manipulations detailed in^32^, one obtains the corrected vector given in Eq. (4).

It can be shown that for functions that are thrice continuously differentiable, the refined vector satisfies

$$\begin{aligned}y_{kt} = \nabla ^2 \phi _{k+1} s_k + O(\Vert s_k\Vert ^3),\end{aligned}$$

while the standard gradient difference satisfies only

$$\begin{aligned}y_k = \nabla ^2 \phi _{k+1} s_k + O(\Vert s_k\Vert ^2).\end{aligned}$$

Thus, $y_{kt}$ achieves third-order approximation accuracy, providing a more precise characterization of the local curvature structure of the objective function.

To extend the applicability of the proposed methodology to large-scale optimization, this paper integrates the framework with a stochastic optimization strategy, resulting in a stochastic three term conjugate gradient algorithm based on third order curvature approximation. The iterative scheme of the TASCG algorithm is presented in Algorithm 1.

**Algorithm 1:**
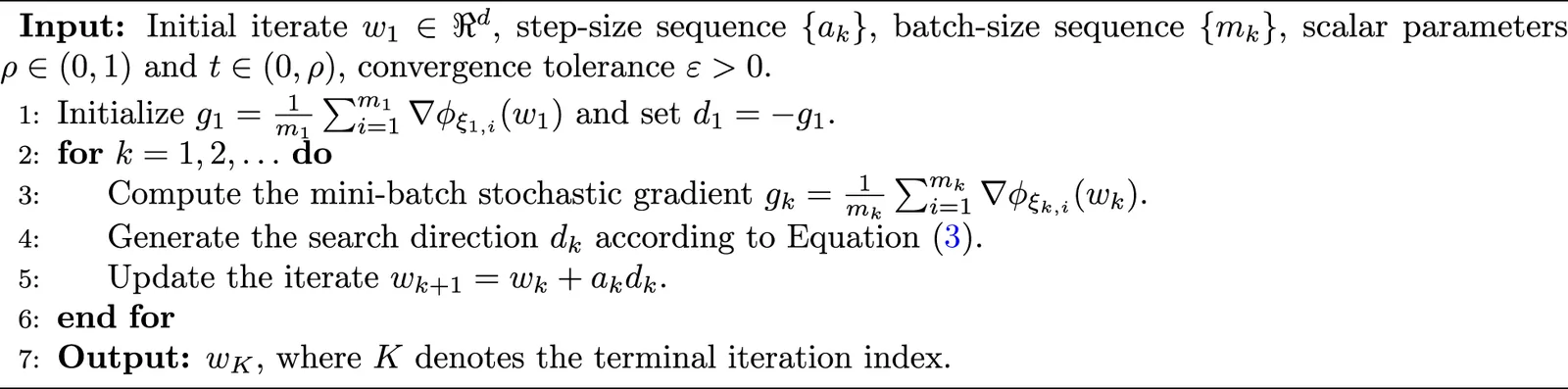
TASCG: stochastic three-term conjugate gradient method with third-order curvature approximation.

In each iteration, the TASCG algorithm requires only the evaluation of a mini-batch stochastic gradient $g_k$, while leveraging historical information to construct a refined curvature vector $y_{k-1,t}$. This design allows incorporation of a third order tensor curvature correction with negligible computational overhead.

### The TASCG framework with integrated variance reduction

The SVRG method improves algorithmic performance by explicitly reducing the variance inherent in stochastic gradient estimates. In contrast to standard Stochastic Gradient Descent, a key advantage of the SVRG framework is that it eliminates the need for a decaying step size sequence $a_k$. A comparatively large step size can be maintained during the optimization process, which accelerates convergence.

The central idea of SVRG is the introduction of a variance reduction mechanism designed to reduce the dispersion of gradient estimates at each iteration. Specifically, SVRG computes a stochastic gradient and then corrects it using the difference between the stochastic gradient evaluated at a previous reference point and the full gradient at that same point. The resulting variance-reduced gradient estimator is given by

$$\begin{aligned} g_{i}^{k+1} = \nabla \phi _{B}(w_{i}^{k+1}) - \nabla \phi _{B}(\tilde{w}_k) + \nabla \phi (\tilde{w}_k),\end{aligned}$$

where $\nabla \phi _{B}(w_{i}^{k+1}) = \frac{1}{|B|} \sum _{i \in B} \nabla \phi _i(w_{i}^{k+1})$ denotes the stochastic gradient computed over a randomly sampled mini-batch $B \subset \{1,2,\dots ,\mathcal {W}\}$ at the *(k+1)*-th outer iteration; $\nabla \phi _{B}(\tilde{w}_k)$ is the stochastic gradient evaluated on the same mini-batch *B* at the reference point $\tilde{w}_k$ and $\nabla \phi (\tilde{w}_k)$ is the full gradient evaluated at this reference point.

To further improve both efficiency and optimization accuracy, this paper proposes an integrated framework that combines Algorithm 1 with variance reduction techniques. This integration preserves the advantages of the stochastic three-term conjugate gradient method while improving gradient estimation accuracy through explicit variance control. The resulting method enhances algorithmic stability, accelerates convergence, and improves the quality of the final solution.

**Algorithm 2:**
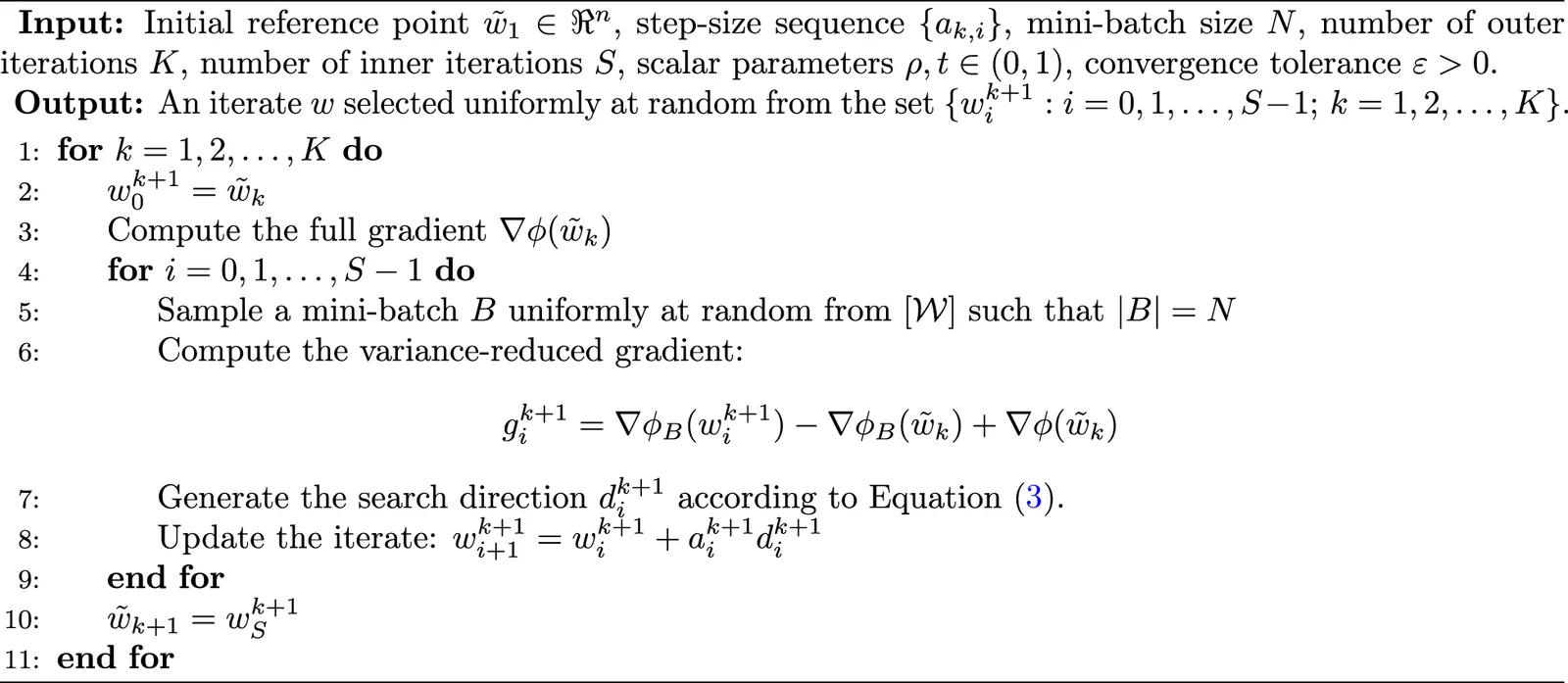
TASCG-VR: TASCG algorithm augmented with variance reduction.

## Convergence analysis and iteration complexity of the algorithm

This section establishes the theoretical guarantees underlying the stochastic three-term conjugate gradient algorithm augmented with a third-order curvature approximation. Under standard assumptions of Lipschitz continuity and bounded gradient variance, we rigorously analyze both its global convergence properties and iteration complexity.

### Assumption 1

Each component function $\phi _i: \Re ^d \rightarrow \Re$ is continuously differentiable, and its gradient is Lipschitz continuous; that is, there exist constants $L_i > 0$ such that, for any $x, y \in \Re ^d$ and all *i = 1, … , n*,

5 $$\begin{aligned} \Vert \nabla \phi _i(x) - \nabla \phi _i(y)\Vert \le L_i \Vert x - y\Vert . \end{aligned}$$

where $$L = \max _{1 \le i \le n} L_i$$.

### Assumption 2

There exists a constant $\sigma \ge 0$ such that, for any iterate $w_k$, the stochastic gradient $g_k$ satisfies

$$\begin{aligned} \mathbb {E}_{\xi _k}\left[ g_k \mid w_k\right] = \nabla \phi (w_k), \qquad \mathbb {E}_{\xi _k}\left[ \Vert g_k - \nabla \phi (w_k)\Vert ^2 \mid w_k\right] \le \sigma ^2,\end{aligned}$$

where $\{\xi _k\}_{k=1}^\infty$ are independent samples, and each $\xi _k$ is independent of $\{w_j\}_{j=1}^{k-1}$. In particular, if

$$\begin{aligned}g_k = \frac{1}{m_k} \sum _{i=1}^{m_k} g_k^{(i)}\end{aligned}$$

is the average of $m_k$ independent and identically distributed stochastic gradients, then the variance bound improves to

6 $$\begin{aligned} \mathbb {E}\left[ \Vert g_k - \nabla \phi (w_k)\Vert ^2 \mid w_k\right] \le \frac{\sigma ^2}{m_k}. \end{aligned}$$

Under the aforementioned assumptions, the direction $d_k$ generated by the algorithm is shown to satisfy two pivotal properties: the sufficient descent condition and boundedness. These properties form the foundation for the subsequent convergence analysis, and the corresponding conclusions are summarized in the following lemma.

### Lemma 1

*Given that the search direction* $d_k$ *is determined by an algorithm with parameters satisfying* *0< t< ρ < 1*, *there exist constants* $C_1 > 0$ *and* $C_2 > 0$ *such that, for all* *k ≥ 0*, *the following conditions hold*:

$$\begin{aligned} g_k^T d_k \le -C_1 \Vert g_k\Vert ^2, \qquad \Vert d_k\Vert \le C_2 \Vert g_k\Vert . \end{aligned}$$

*In particular, these constants may be defined as*

$$\begin{aligned} C_1 = \min \{1 - \rho - t,\ \rho - t\}, \qquad C_2 = 2 + \rho + t. \end{aligned}$$

### Proof

For *k = 0*, we have $d_0 = -g_0$, and the conclusion holds trivially. For *k ≥ 1*, the definition of the search direction yields

$$\begin{aligned} d_k = -g_k + \beta _k^{\text {NE}+} d_{k-1} - \rho \frac{g_k^T d_{k-1}}{\delta _k} y_{kt}, \end{aligned}$$

where $$y_k = \nabla \phi _k - \nabla \phi _{k-1}$$, $$s_k = w_k - w_{k-1}$$, $$\delta _k = \max \{\Vert d_{k-1}\Vert \Vert y_{kt}\Vert , \Vert d_{k-1}\Vert \Vert s_k\Vert \}$$, and

$$\begin{aligned} \beta _k^{\text {NE}+} = \max \left\{ \frac{g_k^T y_{kt}}{\delta _k}, 0\right\} - \frac{t g_k^T s_k}{\delta _k}. \end{aligned}$$

Computing the inner product yields

$$\begin{aligned} g_k^T d_k = -\Vert g_k\Vert ^2 + \beta _k^{\text {NE}+}(g_k^T d_{k-1}) - \rho \frac{(g_k^T d_{k-1})(g_k^T y_{kt})}{\delta _k}. \end{aligned}$$

1. Sufficient descent property. We proceed by considering two cases based on the sign of $\dfrac{g_k^T y_{kt}}{\delta _k}$.

Case 1. $\dfrac{g_k^T y_{kt}}{\delta _k} \le 0$. In this case, $\max \{\dfrac{g_k^T y_{kt}}{\delta _k},0\} = 0$, and $g_k^T d_k$ reduces to

$$\begin{aligned} g_k^T d_k = -\Vert g_k\Vert ^2 - \frac{t g_k^T s_k}{\delta _k} (g_k^T d_{k-1}) - \rho \frac{(g_k^T d_{k-1})(g_k^T y_{kt})}{\delta _k}. \end{aligned}$$

Since $-\dfrac{t g_k^T s_k}{\delta _k} (g_k^T d_{k-1}) \le t \Vert g_k\Vert ^2$ and the third term is nonpositive, we obtain

$$\begin{aligned} g_k^T d_k \le -(1 - t - \rho )\Vert g_k\Vert ^2. \end{aligned}$$

Case 2. $\dfrac{g_k^T y_{kt}}{\delta _k} > 0$. In this case,

$$\begin{aligned} g_k^T d_k = -\Vert g_k\Vert ^2 + (1-\rho )\frac{(g_k^T d_{k-1})(g_k^T y_{kt})}{\delta _k} - \frac{t g_k^T s_k}{\delta _k} (g_k^T d_{k-1}) \le -(\rho - t)\Vert g_k\Vert ^2. \end{aligned}$$

Combining both cases and setting $C_1 = \min \{1 - \rho - t,\ \rho - t\} > 0$ yields the sufficient descent property.

2. Boundedness. By the triangle inequality,

$$\begin{aligned} \Vert d_k\Vert \le \Vert g_k\Vert + \Vert \beta _k^{\text {NE}+} d_{k-1}\Vert + \left\| \rho \frac{g_k^T d_{k-1}}{\delta _k} y_{kt} \right\| . \end{aligned}$$

The latter two terms admit the following estimates:

$$\begin{aligned} & \Vert \beta _k^{\text {NE}+} d_{k-1}\Vert \le \left( \left| \frac{g_k^T y_{kt}}{\delta _k}\right| + \frac{t |g_k^T s_k|}{\delta _k} \right) \Vert d_{k-1}\Vert \le (1 + t)\Vert g_k\Vert , \\ & \left\| \rho \frac{g_k^T d_{k-1}}{\delta _k} y_{kt} \right\| \le \rho \Vert g_k\Vert . \end{aligned}$$

Substituting these bounds yields $\Vert d_k\Vert \le (2 + \rho + t)\Vert g_k\Vert$. Hence, taking $C_2 = 2 + \rho + t$ satisfies the requirement. *□*

Lemma 1 establishes the sufficient descent property and boundedness of the search direction in the deterministic setting. In practical implementations, however, the direction $d_k$ depends on the stochastic gradient $g_k$. To extend these properties to the stochastic setting while accounting for the effects of randomness, the following lemma provides a one-step descent inequality in expectation.

### Lemma 2

*Let* $\phi : \Re ^n \rightarrow \Re$ *be an* *L*-*smooth function, and suppose that Assumptions* 1*and* 2*hold. Then the following inequality is valid*:

$$\begin{aligned} \mathbb {E}[\phi (w_{k+1})] \le \mathbb {E}[\phi (w_k)] - a_k L^* \mathbb {E}[\Vert \nabla \phi (w_k)\Vert ^2] + \frac{C_3\sigma ^2}{2m} a_k^2. \end{aligned}$$

where $0 < a_k \le \dfrac{C_1 - 2L^*}{L C_2^2}$, $C_3= \frac{C_2^2 + C_1^2 - 2L^* C_1}{C_1}$ and *m* is a constant.

### Proof

By the *L*-smoothness of *ϕ*, for any $x, y \in \mathbb {R}^n$ we have

$$\begin{aligned} \phi (y) \le \phi (x) + \nabla \phi (x)^\textsf{T}(y - x) + \frac{L}{2}\Vert y - x\Vert ^2. \end{aligned}$$

Setting $x = w_k$ and $y = w_{k+1} = w_k + a_k d_k$ yields

7 $$\begin{aligned} \phi (w_{k+1}) \le \phi (w_k) + a_k \nabla \phi (w_k)^\textsf{T} d_k + \frac{L}{2} a_k^2 \Vert d_k\Vert ^2. \end{aligned}$$

Taking expectations on both sides and applying the law of total expectation, we obtain

8 $$\begin{aligned} \mathbb {E}[\phi (w_{k+1})] \le \mathbb {E}[\phi (w_k)] + a_k \mathbb {E}[\nabla \phi (w_k)^\textsf{T} d_k] + \frac{L}{2} a_k^2 \mathbb {E}[\Vert d_k\Vert ^2]. \end{aligned}$$

To handle the inner product term, we use the identity

$$\begin{aligned} \nabla \phi (w_k)^\textsf{T} d_k = g_k^\textsf{T} d_k - (g_k - \nabla \phi (w_k))^\textsf{T} d_k. \end{aligned}$$

By Lemma 1, the direction satisfies the almost sure sufficient descent property

$$\begin{aligned} g_k^\textsf{T} d_k \le -C_1 \Vert g_k\Vert ^2, \end{aligned}$$

and the boundedness property $\Vert d_k\Vert \le C_2 \Vert g_k\Vert$, where $C_1, C_2 > 0$. Applying the Cauchy–Schwarz inequality to the cross term gives

$$\begin{aligned} \bigl |(g_k - \nabla \phi (w_k))^\textsf{T} d_k\bigr | \le C_2 \Vert g_k - \nabla \phi (w_k)\Vert \Vert g_k\Vert . \end{aligned}$$

Using Young’s inequality with $\epsilon = \frac{C_1}{C_2}$, we obtain

$$\begin{aligned} C_2 \Vert g_k - \nabla \phi (w_k)\Vert \Vert g_k\Vert \le \frac{C_1}{2} \Vert g_k\Vert ^2 + \frac{C_2^2}{2C_1} \Vert g_k - \nabla \phi (w_k)\Vert ^2. \end{aligned}$$

Combining the estimates above yields

9 $$\begin{aligned} \nabla \phi (w_k)^\textsf{T} d_k \le -\frac{C_1}{2} \Vert g_k\Vert ^2 + \frac{C_2^2}{2C_1} \Vert g_k - \nabla \phi (w_k)\Vert ^2. \end{aligned}$$

Taking expectations and invoking Assumption 2, we have

$$\begin{aligned} \mathbb {E}[\nabla \phi (w_k)^\textsf{T} d_k] \le -\frac{C_1}{2} \mathbb {E}[\Vert g_k\Vert ^2] + \frac{C_2^2}{2C_1} \cdot \frac{\sigma ^2}{m_k}. \end{aligned}$$

Since $\mathbb {E}[\Vert g_k\Vert ^2] \ge \mathbb {E}[\Vert \nabla \phi (w_k)\Vert ^2]$ and $-\frac{C_1}{2} < 0$, it follows that

10 $$\begin{aligned} \mathbb {E}[\nabla \phi (w_k)^\textsf{T} d_k] \le -\frac{C_1}{2} \mathbb {E}[\Vert \nabla \phi (w_k)\Vert ^2] + \frac{C_2^2}{2C_1} \cdot \frac{\sigma ^2}{m_k}. \end{aligned}$$

For the squared norm of the direction, the boundedness property together with Assumption 2 gives

11 $$\begin{aligned} \mathbb {E}[\Vert d_k\Vert ^2] \le C_2^2 \mathbb {E}[\Vert g_k\Vert ^2] \le C_2^2 \Bigl ( \mathbb {E}[\Vert \nabla \phi (w_k)\Vert ^2] + \frac{\sigma ^2}{m_k} \Bigr ). \end{aligned}$$

Substituting (10) and (11) into (8) we obtain

12 $$\begin{aligned} \mathbb {E}[\phi (w_{k+1})] \le \mathbb {E}[\phi (w_k)] - a_k \Bigl ( \frac{C_1}{2} - \frac{L C_2^2}{2} a_k \Bigr ) \mathbb {E}[\Vert \nabla \phi (w_k)\Vert ^2] + a_k \frac{C_2^2}{2C_1} \frac{\sigma ^2}{m_k} + \frac{L C_2^2 a_k^2}{2} \frac{\sigma ^2}{m_k}. \end{aligned}$$

Now choose $m_k = \lceil m / a_k \rceil$ so that $\frac{1}{m_k} \le \frac{a_k}{m}$. Substituting this bound into the noise terms of (12) and grouping constants, we get

13 $$\begin{aligned} \mathbb {E}[\phi (w_{k+1})] \le \mathbb {E}[\phi (w_k)] - a_k \Bigl ( \frac{C_1}{2} - \frac{L C_2^2}{2} a_k \Bigr ) \mathbb {E}[\Vert \nabla \phi (w_k)\Vert ^2] + \frac{(C_2^2+L C_2^2 C_1 a_k)\sigma ^2}{2m C_1} a_k^2. \end{aligned}$$

To ensure a strictly positive descent coefficient, we introduce a constant $L^* > 0$ and impose the condition $\frac{C_1}{2} - \frac{L C_2^2 a_k}{2} \ge L^*,$ which is equivalent to $a_k \le \frac{C_1 - 2L^*}{L C_2^2}$. Substituting this bound into (13) yields the desired inequality:

$$\begin{aligned} \mathbb {E}[\phi (w_{k+1})] \le \mathbb {E}[\phi (w_k)] - a_k L^* \mathbb {E}[\Vert \nabla \phi (w_k)\Vert ^2] + \frac{C_3\sigma ^2}{2m} a_k^2, \end{aligned}$$

where $$C_3 = \frac{C_2^2 + C_1^2 - 2L^* C_1}{C_1}$$. *□*

Lemma 2 establishes a recursive relationship linking the expected objective decrease, the gradient norm, and the variance term, which provides the foundation for the subsequent convergence analysis. It is worth noting that the step-size condition $\sum _{k=1}^\infty \alpha _k^2<\infty$ plays a key role in bounding the expected function values.Such a condition is widely adopted in the convergence analysis of stochastic gradient descent for nonconvex optimization^33^, where similar boundedness results of the form $\mathbb {E}[\phi (w_k)]\le M_\phi$ have been established to ensure the stability of the iterative sequence.Based on this inequality, the following global convergence result can be established by constructing an appropriate auxiliary sequence.

### Theorem 1

*Suppose that Assumptions* 1*and* 2*hold. Let the step-size sequence*$\{a_k\}$ *satisfy* $\sum _{k=1}^\infty a_k = \infty$ *and* $\sum _{k=1}^\infty a_k^2 < \infty$. *Then the sequence* $\{w_k\}$ *generated by the stochastic gradient descent algorithm* satisfies

$$\begin{aligned} \liminf _{k \rightarrow \infty } \Vert \nabla \phi (w_k)\Vert = 0, \end{aligned}$$

*and there exists a constant* $M_\phi > 0$ *such that* $\mathbb {E}[\phi (w_k)] \le M_\phi$ *holds for all* *k ≥ 1*.

### Proof

Define $\Phi _k := \phi (w_k) + \frac{C_3\sigma ^2}{2m} \sum _{i=k}^\infty a_i^2$. From Lemma 2, we obtain

$$\begin{aligned} \mathbb {E}[\phi (w_{k+1}) \mid \mathcal {F}_k] \le \phi (w_k) - a_k L^* \Vert \nabla \phi (w_k)\Vert ^2 + \frac{C_3\sigma ^2}{2m} a_k^2. \end{aligned}$$

Consequently,

$$\begin{aligned} \begin{aligned} \mathbb {E}[\Phi _{k+1} \mid \mathcal {F}_k]&= \mathbb {E}[\phi (w_{k+1}) \mid \mathcal {F}_k] + \frac{C_3\sigma ^2}{2m} \sum _{i=k+1}^\infty a_i^2 \\&\le \phi (w_k) - a_k L^* \Vert \nabla \phi (w_k)\Vert ^2 + \frac{C_3\sigma ^2}{2m} a_k^2 + \frac{C_3\sigma ^2}{2m} \sum _{i=k+1}^\infty a_i^2 \\&= \Phi _k - a_k L^* \Vert \nabla \phi (w_k)\Vert ^2. \end{aligned} \end{aligned}$$

Let $\Omega _k := a_k L^* \Vert \nabla \phi (w_k)\Vert ^2 \ge 0$. Then $\mathbb {E}[\Phi _{k+1} \mid \mathcal {F}_k] \le \Phi _k - \Omega _k$. Since $\phi (w) \ge \phi ^{\text {low}}$ and the summation term is nonnegative, it follows that $\Phi _k \ge \phi ^{\text {low}}$. Consequently, $\{\Phi _k - \phi ^{\text {low}}\}$ is a nonnegative supermartingale. By the martingale convergence theorem, $\Phi _k$ converges almost surely to a finite random variable $\Phi _\infty$, and $\sum _{k=1}^\infty \Omega _k < \infty$ holds almost surely.

From $\sum _{k=1}^\infty \Omega _k < \infty$, i.e., $\sum _{k=1}^\infty a_k L^* \Vert \nabla \phi (w_k)\Vert ^2 < \infty$, together with $L^* > 0$ and $\sum _{k=1}^\infty a_k = \infty$, it follows that $\liminf _{k \rightarrow \infty } \Vert \nabla \phi (w_k)\Vert = 0$. Indeed, if $\liminf _{k \rightarrow \infty } \Vert \nabla \phi (w_k)\Vert > 0$, then there would exist *ε > 0* and an integer *K* such that $\Vert \nabla \phi (w_k)\Vert ^2 \ge \epsilon$ for all *k > K*. This would imply

$$\begin{aligned} \sum _{k=K+1}^\infty a_k L^* \Vert \nabla \phi (w_k)\Vert ^2 \ge L^* \epsilon \sum _{k=K+1}^\infty a_k = \infty , \end{aligned}$$

contradicting the almost sure finiteness of the series.

On the other hand, taking the total expectation of the descent inequality yields $\mathbb {E}[\Phi _{k+1}] \le \mathbb {E}[\Phi _k] - \mathbb {E}[\Omega _k] \le \mathbb {E}[\Phi _k]$. Thus, $\{\mathbb {E}[\Phi _k]\}$ is a monotonically nonincreasing sequence that is bounded below and therefore convergent. Consequently,

$$\begin{aligned} \mathbb {E}[\phi (w_k)] \le \mathbb {E}[\Phi _k] \le \mathbb {E}[\Phi _1] =: M_\phi . \end{aligned}$$

*□*

Theorem 1 guarantees only that the limit inferior of the gradient norm is zero. To obtain convergence of the entire sequence, an additional uniform boundedness condition on the second moment of the stochastic gradient is required. Under this condition, the gradient norm can be shown to converge to zero along the whole sequence.

### Theorem 2

(Stronger Convergence). *Under the conditions of Theorem*1, *suppose additionally that there exists a constant* $M_g > 0$ *such that* $\mathbb {E}[\Vert g_k\Vert ^2 \mid w_k] \le M_g$. Then

$$\begin{aligned} \lim _{k \rightarrow \infty } \Vert \nabla \phi (w_k)\Vert = 0. \end{aligned}$$

### Proof

We proceed by contradiction. Suppose that the conclusion does not hold. Then there exist *ε > 0* and two infinite strictly increasing integer sequences $\{m_i\}$ and $\{n_i\}$ with $m_i< n_i < m_{i+1}$ such that

$$\begin{aligned} \Vert \nabla \phi (w_{m_i})\Vert \ge 2\epsilon , \quad \Vert \nabla \phi (w_{n_i})\Vert < \epsilon , \quad \text {and} \quad \Vert \nabla \phi (w_k)\Vert \ge \epsilon \quad \text {for all } k \in (m_i, n_i). \end{aligned}$$

From the proof of Theorem 1, we have $\sum _{k=1}^\infty a_k \Vert \nabla \phi (w_k)\Vert ^2 < \infty$ almost surely. Consequently,

$$\begin{aligned} \infty > \sum _{k=1}^\infty a_k \Vert \nabla \phi (w_k)\Vert ^2 \ge \sum _{i=1}^\infty \sum _{k=m_i}^{n_i-1} a_k \Vert \nabla \phi (w_k)\Vert ^2 \ge \epsilon ^2 \sum _{i=1}^\infty \sum _{k=m_i}^{n_i-1} a_k, \end{aligned}$$

which implies that $\sum _{k=m_i}^{n_i-1} a_k \rightarrow 0$ as $i \rightarrow \infty$.

We now examine the variation of the iterates $w_k$ over the interval $[m_i, n_i]$. By the update formula $w_{k+1} = w_k + a_k d_k$ and the bound $\Vert d_k\Vert \le C_2 \Vert g_k\Vert$, applying Jensen’s inequality yields

$$\begin{aligned} \mathbb {E}[\Vert w_{k+1} - w_k\Vert \mid \mathcal {F}_k] \le a_k C_2 \mathbb {E}[\Vert g_k\Vert \mid \mathcal {F}_k] \le a_k C_2 \sqrt{\mathbb {E}[\Vert g_k\Vert ^2 \mid \mathcal {F}_k]} \le a_k C_2 \sqrt{M_g}. \end{aligned}$$

Taking expectations and summing over the interval gives

$$\begin{aligned} \mathbb {E}[\Vert w_{n_i} - w_{m_i}\Vert ] \le \sum _{k=m_i}^{n_i-1} \mathbb {E}[\Vert w_{k+1} - w_k\Vert ] \le C_2 \sqrt{M_g} \sum _{k=m_i}^{n_i-1} a_k. \end{aligned}$$

Since $\sum _{k=m_i}^{n_i-1} a_k \rightarrow 0$, it follows that $\mathbb {E}[\Vert w_{n_i} - w_{m_i}\Vert ] \rightarrow 0$. By Markov’s inequality, $\Vert w_{n_i} - w_{m_i}\Vert \rightarrow 0$ in probability.

By the Lipschitz continuity of $\nabla \phi$, we obtain $\Vert \nabla \phi (w_{n_i}) - \nabla \phi (w_{m_i})\Vert \rightarrow 0$ in probability. However, by construction of the sequences, $\Vert \nabla \phi (w_{m_i})\Vert \ge 2\epsilon$ and $\Vert \nabla \phi (w_{n_i})\Vert < \epsilon$, which implies that $\Vert \nabla \phi (w_{n_i}) - \nabla \phi (w_{m_i})\Vert \ge \epsilon$ almost surely. This yields a contradiction. Therefore, the initial supposition is false, and the theorem is established. *□*

The preceding theorem characterizes the asymptotic behavior of the algorithm. To obtain a quantitative assessment of the convergence rate over a finite number of iterations, this section further analyzes the iteration complexity. We first consider the case in which the step size decays polynomially.

### Theorem 3

*Suppose that Assumptions* 1*and* 2*hold, and let the step size be chosen as*

$$\begin{aligned} a_k = \dfrac{L^*}{C_3}k^{-\beta }, \quad \beta \in (0.5, 1), \end{aligned}$$

*with* *m* *kept constant. Then*

$$\begin{aligned} \frac{1}{N} \sum _{k=1}^N \mathbb {E}[\Vert \nabla \phi (w_k)\Vert ^2] \le \frac{C_3(M_\phi - \phi ^{\text {low}})}{(L^*)^2} N^{\beta -1} + \frac{\sigma ^2(N^{-\beta } - 2\beta N^{\beta -1})}{(1-2\beta )m}, \end{aligned}$$

*where* $$M_\phi = \sup _{k \ge 1} \mathbb {E}[\phi (w_k)]$$.

### Proof

From Lemma 2, we obtain

$$\begin{aligned} \mathbb {E}[\phi (w_{k+1})] \le \mathbb {E}[\phi (w_k)] - a_k L^* \mathbb {E}[\Vert \nabla \phi (w_k)\Vert ^2] + \frac{C_3\sigma ^2}{2m} a_k^2. \end{aligned}$$

Rearranging yields

$$\begin{aligned} a_k L^* \mathbb {E}[\Vert \nabla \phi (w_k)\Vert ^2] \le \mathbb {E}[\phi (w_k)] - \mathbb {E}[\phi (w_{k+1})] + \frac{C_3\sigma ^2}{2m} a_k^2. \end{aligned}$$

Summing over *k = 1, … , N* gives

$$\begin{aligned} L^* \sum _{k=1}^N a_k \mathbb {E}[\Vert \nabla \phi (w_k)\Vert ^2] \le \mathbb {E}[\phi (w_1)] - \mathbb {E}[\phi (w_{N+1})] + \frac{C_3\sigma ^2}{2m} \sum _{k=1}^N a_k^2. \end{aligned}$$

Since $\mathbb {E}[\phi (w_{N+1})] \ge \phi ^{\text {low}}$ and $\mathbb {E}[\phi (w_1)] \le M_\phi$, we have

$$\begin{aligned} \sum _{k=1}^N a_k \mathbb {E}[\Vert \nabla \phi (w_k)\Vert ^2] \le \frac{M_\phi - \phi ^{\text {low}}}{L^*} + \frac{C_3\sigma ^2}{2mL^*} \sum _{k=1}^N a_k^2. \end{aligned}$$

Substituting $a_k = c k^{-\beta }$ with $c = \dfrac{L^*}{C_3}$, we obtain the bounds

$$\begin{aligned} & \sum _{k=1}^N a_k \ge c \int _1^N x^{-\beta } \, dx = c \frac{N^{1-\beta } - 1}{1-\beta }, \\ & \sum _{k=1}^N a_k^2 = c^2 \sum _{k=1}^N k^{-2\beta } \le c^2 \left( 1 + \int _1^N x^{-2\beta } \, dx\right) \le c^2 \frac{N^{1-2\beta }-2\beta }{1-2\beta }. \end{aligned}$$

Let $$A = \sum _{k=1}^N a_k \mathbb {E}[\Vert \nabla \phi (w_k)\Vert ^2]$$, then

$$\begin{aligned} A \le \frac{M_\phi - \phi ^{\text {low}}}{L^*} + \frac{C_3 \sigma ^2}{2m L^*} \cdot c^2 \frac{N^{1-2\beta }-2\beta }{1-2\beta }. \end{aligned}$$

For sufficiently large *N*, we have $\sum _{k=1}^N a_k \ge c \dfrac{N^{1-\beta }}{2(1-\beta )}$. Consequently,

$$\begin{aligned} \frac{1}{N} \sum _{k=1}^N \mathbb {E}[\Vert \nabla \phi (w_k)\Vert ^2] \le \frac{A}{N \cdot \min _{k \le N} a_k} = \frac{A}{N \cdot c N^{-\beta }} = \frac{A}{c} N^{\beta -1}. \end{aligned}$$

Substituting the upper bound for *A* and $c = \dfrac{L^*}{C_3}$, we arrive at

$$\begin{aligned} \frac{1}{N} \sum _{k=1}^N \mathbb {E}[\Vert \nabla \phi (w_k)\Vert ^2] \le \frac{C_3(M_\phi - \phi ^{\text {low}})}{(L^*)^2} N^{\beta -1} + \frac{\sigma ^2}{(1-2\beta )m} (N^{-\beta } - 2\beta N^{\beta -1}), \end{aligned}$$

which completes the proof. *□*

In contrast to diminishing step sizes, a constant step size combined with a random output strategy can establish finite-step convergence guarantees in nonconvex optimization. The following theorem provides the fundamental estimate under a constant step size regime.

### Theorem 4

*Suppose that Assumptions* 1*and* 2*hold, and let the step size be constant with* $a_k = a$, *where*

$$\begin{aligned} 0 < a \le \frac{C_1 - 2L^*}{L C_2^2}. \end{aligned}$$

*Let* *R* *be a random variable taking values in* $\{1,\dots ,N\}$ *with probability mass function*

$$\begin{aligned} \Pr (R = k) = \frac{1}{N}, \quad k = 1,\dots ,N. \end{aligned}$$

*Then the following inequality holds*:

$$\begin{aligned} \mathbb {E}\bigl [\Vert \nabla \phi (w_R)\Vert ^2\bigr ] \le \frac{\phi (w_1) - \phi ^{\text {low}}}{a L^* N} + \frac{C_3 \sigma ^2}{2m L^*} \cdot a. \end{aligned}$$

### Proof

From Lemma 2, for any *k ≥ 1* we have

$$\begin{aligned} \mathbb {E}[\phi (w_{k+1})] \le \mathbb {E}[\phi (w_k)] - a L^* \mathbb {E}[\Vert \nabla \phi (w_k)\Vert ^2] + \frac{ C_3 \sigma ^2}{2m} a^2. \end{aligned}$$

Summing the above inequality over *k = 1, … , N* yields

$$\begin{aligned} a L^* \sum _{k=1}^N \mathbb {E}[\Vert \nabla \phi (w_k)\Vert ^2] \le \mathbb {E}[\phi (w_1)] - \mathbb {E}[\phi (w_{N+1})] + \frac{C_3 \sigma ^2}{2m} N a^2. \end{aligned}$$

Since $\mathbb {E}[\phi (w_{N+1})] \ge \phi ^{\text {low}}$, it follows that

$$\begin{aligned} \sum _{k=1}^N \mathbb {E}[\Vert \nabla \phi (w_k)\Vert ^2] \le \frac{\mathbb {E}[\phi (w_1)] - \phi ^{\text {low}}}{a L^*} + \frac{C_3 \sigma ^2}{2m L^*} N a. \end{aligned}$$

By the uniform distribution of *R*, we have

$$\begin{aligned} \mathbb {E}\bigl [\Vert \nabla \phi (w_R)\Vert ^2\bigr ] = \frac{1}{N} \sum _{k=1}^N \mathbb {E}[\Vert \nabla \phi (w_k)\Vert ^2], \end{aligned}$$

and substituting the above bound yields the desired conclusion. *□*

The upper bound established in Theorem 4 consists of two terms, and balancing these terms yields the optimal choice of the step size. Based on this analysis, one can derive the minimum number of iterations required to achieve a prescribed accuracy *ε*, along with the corresponding stochastic first-order (SFO) complexity.

### Theorem 5

*Under the conditions of Theorem* 4, *choose*

$$\begin{aligned} a = \sqrt{\frac{2m \bigl (\mathbb {E}[\phi (w_1)] - \phi ^{\text {low}}\bigr )}{C_3 \sigma ^2 N}}, \end{aligned}$$

*and suppose that* *N* *satisfies*

$$\begin{aligned} N \ge \max \left\{ \frac{4\bigl (\mathbb {E}[\phi (w_1)] - \phi ^{\text {low}}\bigr )}{L^* \epsilon },\ \frac{C_3 \sigma ^2}{2m L^* \epsilon } \right\} . \end{aligned}$$

*Then*

$$\begin{aligned} \mathbb {E}\bigl [\Vert \nabla \phi (w_R)\Vert ^2\bigr ] \le \epsilon , \end{aligned}$$

*and the total number of SFO calls is* $N \cdot m = O(\epsilon ^{-2})$.

### Proof

According to Theorem 4, we have

$$\begin{aligned} \mathbb {E}\bigl [\Vert \nabla \phi (w_R)\Vert ^2\bigr ] \le \frac{\phi (w_1) - \phi ^{\text {low}}}{a L^* N} + \frac{C_3 \sigma ^2}{2m L^*} a. \end{aligned}$$

Substituting the expression for *a*, we compute

$$\begin{aligned} & \frac{\phi (w_1) - \phi ^{\text {low}}}{a L^* N} = \sqrt{\frac{\bigl (\phi (w_1) - \phi ^{\text {low}}\bigr )C_3 \sigma ^2}{2m (L^*)^2 N}}, \\ & \frac{C_3\sigma ^2}{2m L^*} a = \sqrt{\frac{\bigl (\phi (w_1) - \phi ^{\text {low}}\bigr ) C_3 \sigma ^2}{2m (L^*)^2 N}}. \end{aligned}$$

Therefore,

$$\begin{aligned} \mathbb {E}\bigl [\Vert \nabla \phi (w_R)\Vert ^2\bigr ] \le 2 \sqrt{\frac{\bigl (\phi (w_1) - \phi ^{\text {low}}\bigr ) C_3 \sigma ^2}{2m (L^*)^2 N}}. \end{aligned}$$

To ensure that the upper bound does not exceed *ε*, it suffices to require

$$\begin{aligned} N \ge \frac{2C_3 \sigma ^2 \bigl (\phi (w_1) - \phi ^{\text {low}}\bigr )}{m (L^*)^2 \epsilon ^2}. \end{aligned}$$

On the other hand, the condition $a \le \dfrac{C_1 - 2L^*}{L C_2^2}$ yields another lower bound for *N*. Combining both bounds gives the condition on *N* stated in the theorem. In this case, the total number of SFO calls is $N \cdot m = O(\epsilon ^{-2})$. *□*

In summary, the global convergence of the proposed stochastic three-term conjugate gradient algorithm has been established under nonconvex conditions, and its SFO complexity is shown to be $\mathcal {O}(\epsilon ^{-2})$, which is on par with the optimal complexity of classical stochastic gradient methods.

### Remark 1

In this section, we focus on the convergence analysis of the algorithmic framework based on a third-order curvature approximation correction. The theoretical analysis combining the stochastic three-term conjugate gradient algorithm with variance reduction techniques is not yet considered. Incorporating variance reduction into the present framework is nontrivial and poses several challenges: (i) The direction update becomes history-dependent, breaking the simple almost-sure descent property $g_k^\top d_k \le -C_1\Vert g_k\Vert ^2$; (ii) The second-moment bound of the stochastic gradient contains an extra term depending on the reference point, i.e., $\mathbb {E}[\Vert g_k^{\textrm{VR}}\Vert ^2] \le \Vert \nabla \phi (w_k)\Vert ^2 + \frac{\sigma ^2}{m_k} + \mathcal {O}(\Vert \nabla \phi (w_{\textrm{ref}})\Vert ^2)$; (iii) The step size $a_k$ and the variance-reduction parameters (e.g., inner-loop length, reference update frequency) are strongly coupled, making it difficult to derive a unified descent inequality. A rigorous analysis would require new technical tools, such as coupled Lyapunov recursions, and is left for future work.

## Numerical experiments

This section evaluates the numerical performance of the TASCG and TASCG-VR algorithms. For the comparison between TASCG and SGD, we adopt a diminishing step-size strategy, specifically setting $a_k = a \cdot k^{-\beta }$ with $\beta \in (0.5, 1)$. Additionally, a constant step-size configuration is employed in the experiments comparing TASCG-VR and SVRG.

### Test models and datasets

This paper selects two representative models to evaluate the adaptability of the proposed algorithms when confronted with the complex geometric landscapes induced by nonconvex loss functions and nonconvex regularizers. Such landscapes are ubiquitous in modern machine learning tasks and frequently cause conventional first-order methods to stall at local minima or saddle points. Moreover, the hyperbolic tangent loss and the sigmoid loss are representative nonconvex loss functions used in classification and regression, respectively, which facilitates a comprehensive assessment of the algorithms’ robustness and the ability to escape saddle points across diverse nonconvex landscapes. Based on this, the performance of the proposed algorithms is evaluated in the following experiments.

Test Model 1: Nonconvex support vector machine.

$$\begin{aligned} \min _{w \in \Re ^n} \frac{1}{n} \sum _{i=1}^{n} \phi _i(w) + \lambda \Vert w\Vert ^2, \end{aligned}$$

where $\phi _i(w) = 1 - \tanh (b_i \langle w, a_i \rangle )$, $b_i \in \{-1, 1\}$ denotes the class label of the sample, and $a_i \in \Re ^n$ represents the corresponding feature vector. This model employs the hyperbolic tangent function to construct a nonconvex loss term, aiming to capture more intricate classification boundaries. Its nonconvex nature places heightened demands on an algorithm’s ability to escape local extrema and saddle points.

Test Model 2: Nonconvex regularized empirical risk minimization with sigmoid loss.

$$\begin{aligned} \min _{w \in \Re ^n} \frac{1}{n} \sum _{i=1}^{n} \frac{1}{1 + \exp (b_i a_i^T w)} + \frac{\lambda }{2} \Vert w\Vert _1^2, \end{aligned}$$

where $a_i \in \Re ^n$ denotes the feature vector and $b_i \in \Re$ represents the corresponding real-valued label. This model adopts the sigmoid function as the loss metric and incorporates a nonconvex regularizer in the form of the squared $\ell _1$-norm, aiming to induce sparsity while simultaneously introducing nonconvex geometric structure. It enables examination of the algorithm’s robustness and adaptability in nonsmooth, nonconvex optimization settings.

Four widely used benchmark datasets are used in the experiments: Adult, Covtype, Sonar, and Splice. The dimensionality and sample size of each dataset are summarized in Table 1. They span a broad spectrum of scenarios from low-dimensional, small-sample cases to high-dimensional, large-sample regimes, supporting a comprehensive evaluation of the algorithms’ generalization performance and scalability. The optimization is initialized at the zero vector. All code is implemented in MATLAB R2022b and run on a computer with a 1.80 GHz Radeon R7 processor and 16 GB of memory, ensuring consistency of the experimental environment and reproducibility of results.

**Table 1:** Datasets.

Dataset	Training samples (*n*)	Dimension (*d*)
Adult	32,562	123
Covtype	581,012	54
Sonar	208	60
Splice	1000	60

### Numerical experiment

**Fig. 1:**
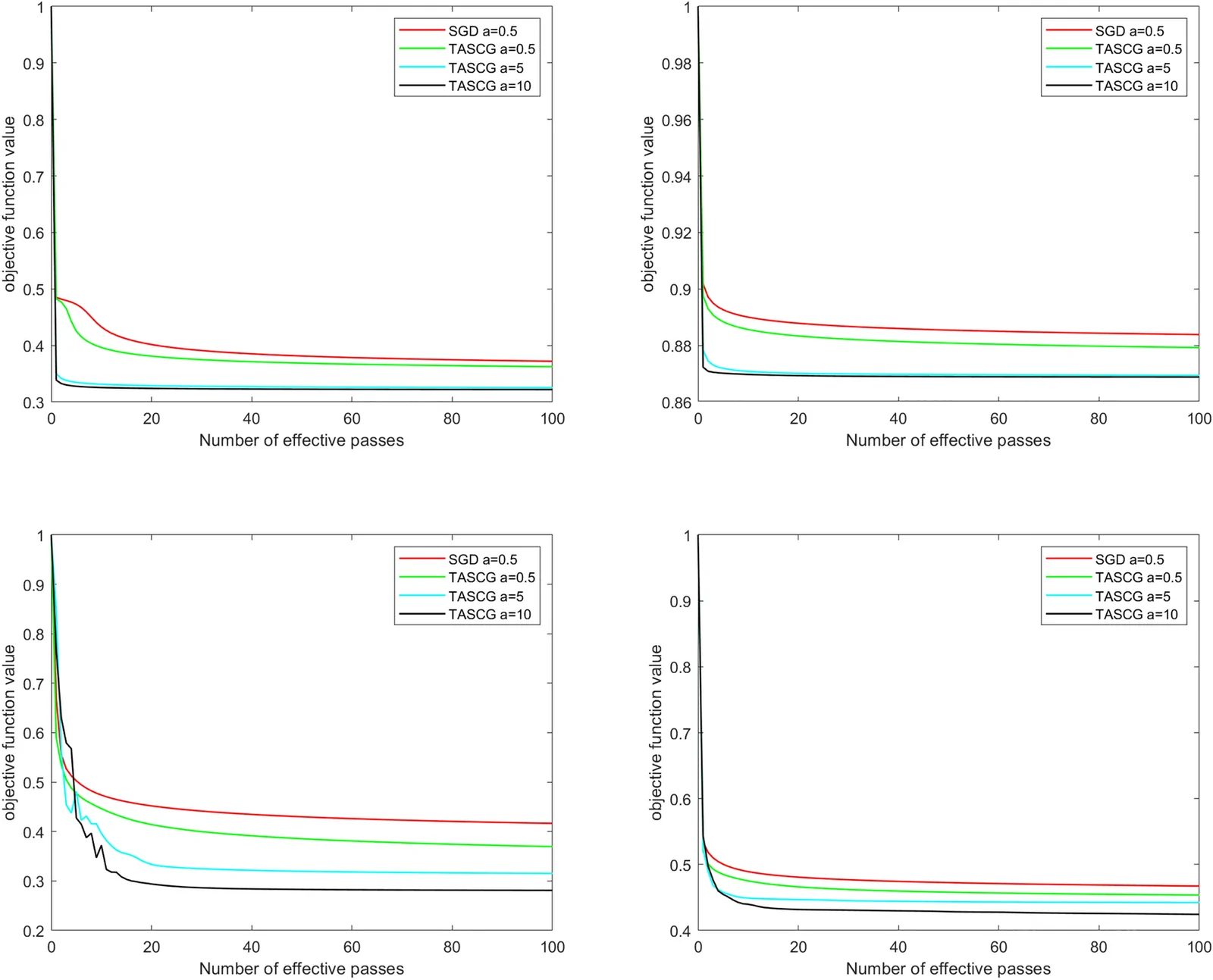
Model 1: performance comparison between TASCG and standard SGD on various datasets.

**Fig. 2:**
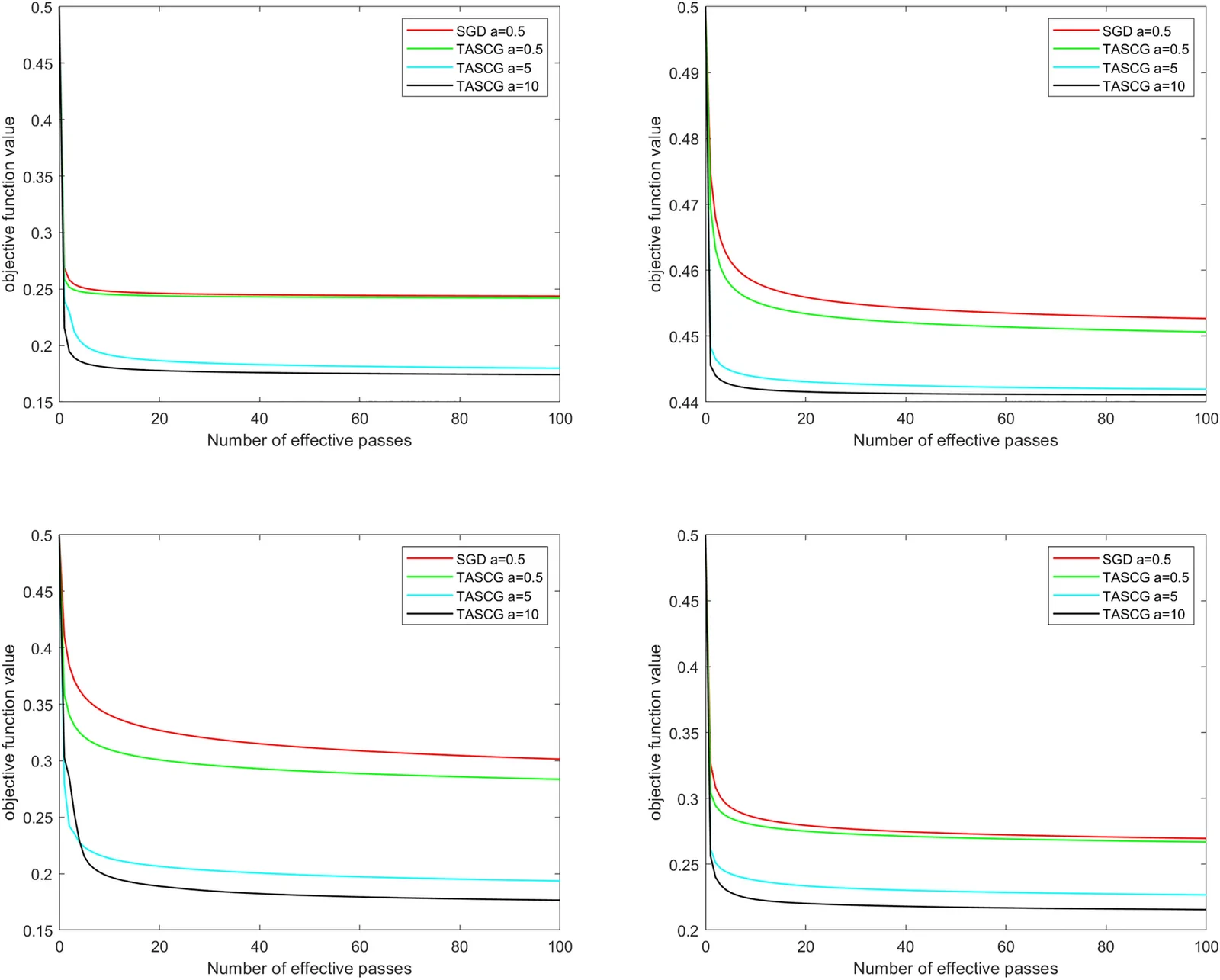
Model 2: performance comparison between TASCG and standard SGD on various datasets.

**Fig. 3:**
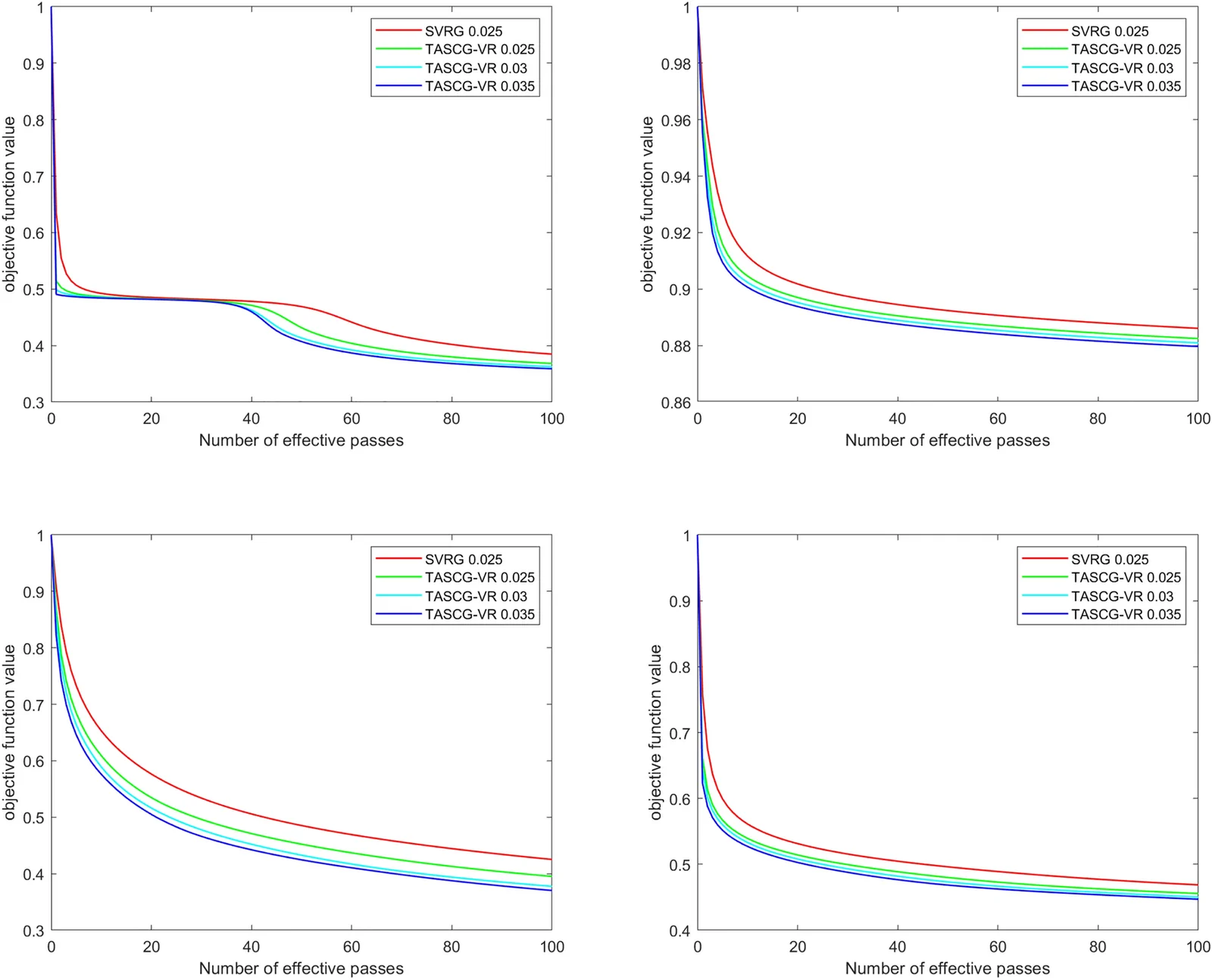
Model 1: performance comparison between TASCG-VR and standard SVRG on various datasets.

**Fig. 4:**
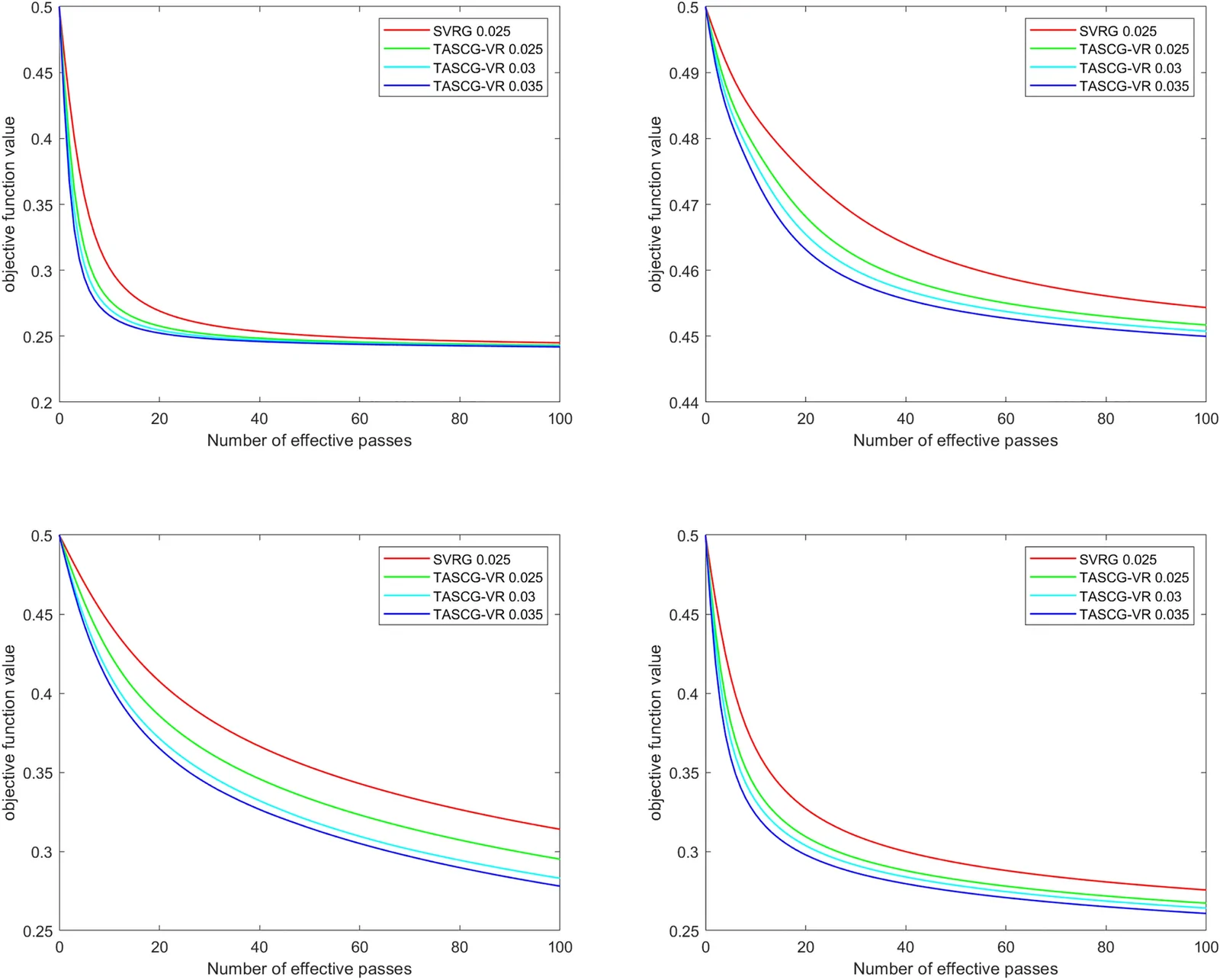
Model 2: performance comparison between TASCG-VR and standard SVRG on various datasets.

**Fig. 5:**
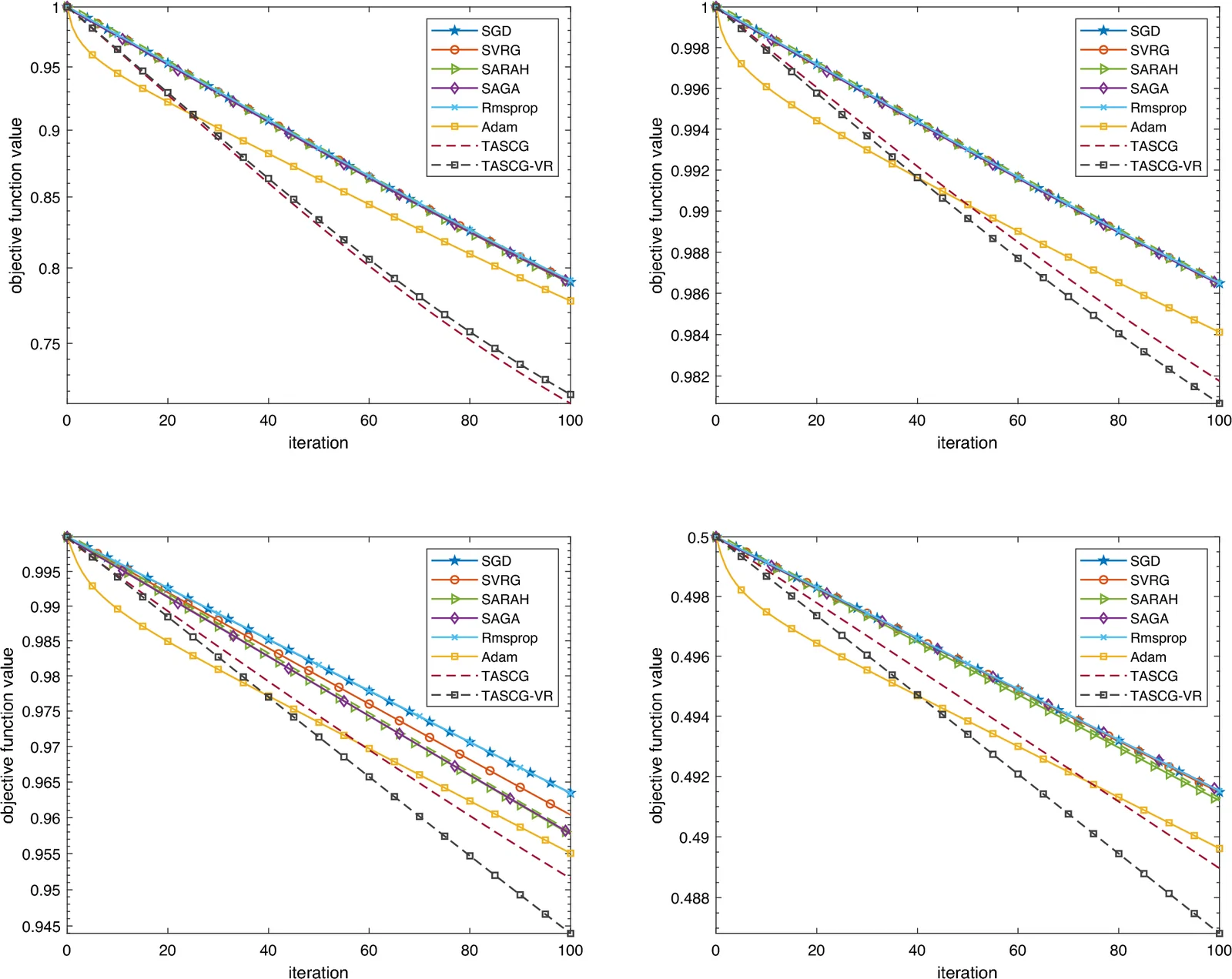
Convergence comparison of different optimization algorithms on Model 1.

**Fig. 6:**
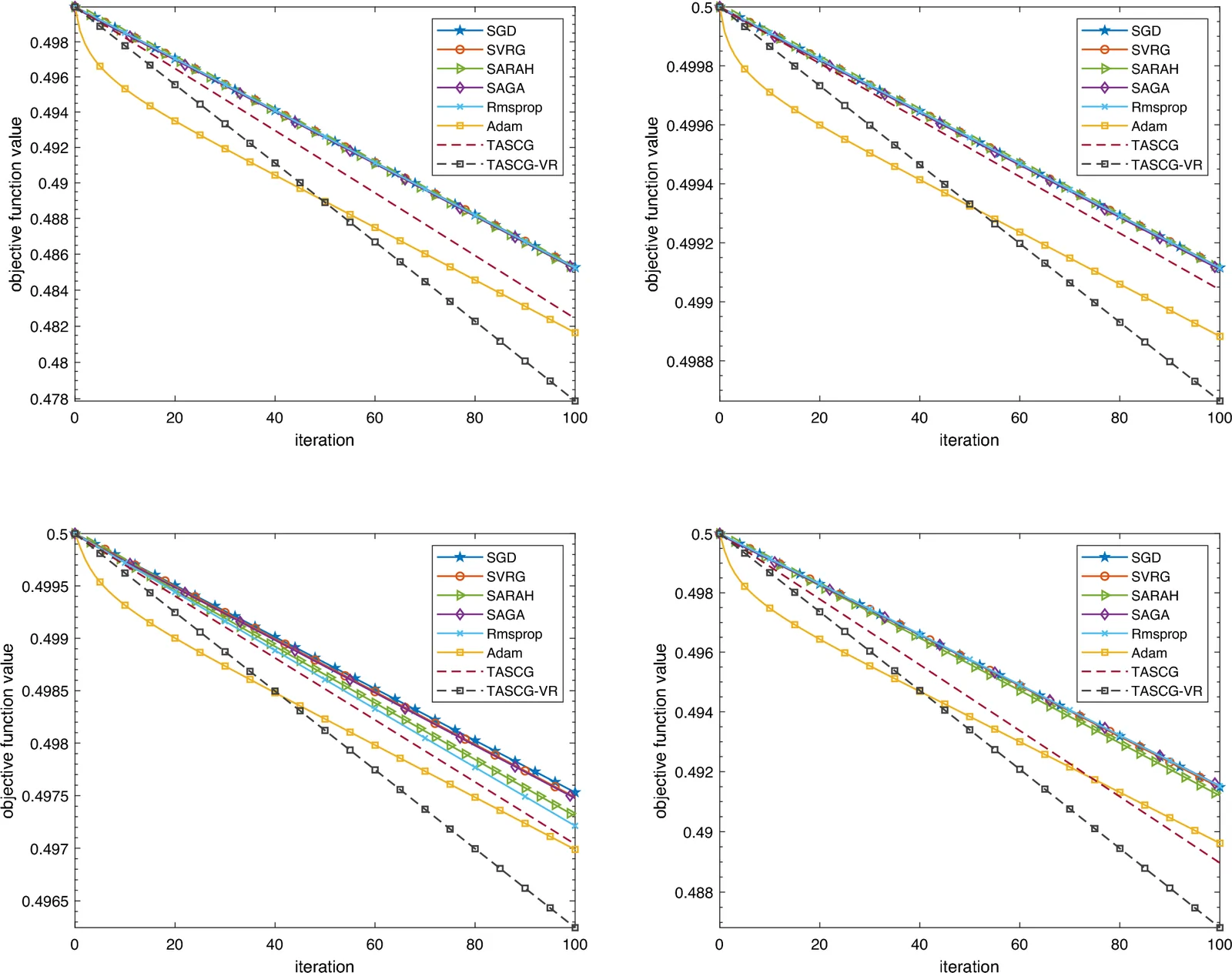
Convergence comparison of different optimization algorithms on Model 2.

In this section, we conduct a comprehensive suite of numerical experiments to evaluate the performance of the TASCG and TASCG-VR algorithms on nonconvex support vector machine models and nonconvex empirical risk minimization problems. The experimental settings are configured uniformly as follows: the mini-batch size is set to $|N_k| = \sqrt{n}$; the step size for Stochastic Gradient Descent (SGD) is fixed at $0.5k^{-0.9}$; the step sizes for the TASCG algorithm are set to $0.5k^{-0.9}$, $5k^{-0.9}$, and $10k^{-0.9}$; the regularization parameter is chosen as $\lambda = 10^{-5}$; the parameters governing the search direction $d_k$ are specified as *t = 0.1* and *ρ = 0.8*; and the initial iterate is taken to be the zero vector. The convergence behavior of each algorithm under the prescribed experimental conditions is assessed by examining the evolution of the objective function value with respect to the iteration count.

As illustrated in Figs. 1 and 2, the experimental results are arrayed-proceeding from left to right-across the four benchmark datasets. In the aggregate, the TASCG algorithm exhibits convergence performance commensurate with that of SGD across both categories of test problems under examination. Within the nonconvex SVM framework, when an identical step-size regime is enforced, TASCG consistently and significantly outperforms SGD on all datasets. Upon escalating the step-size beyond the threshold of $5k^{-0.9}$, performance improvements on the large-sample datasets-namely, Adult and Covtype-evince a tendency toward saturation, yet they remain demonstrably superior to those attained under more conservative step-size configurations. This finding corroborates the premise that a judicious augmentation of the step-size can effectively expedite convergence.

In contradistinction, for the small-sample datasets Sonar and Splice, the imposition of a more aggressive step-size engenders pronounced volatility during the incipient phase of the iterative process, which subsequently yields to a more quiescent and orderly descent. This phenomenon is attributable to the heightened stochasticity inherent in gradient estimation during the initial iterations, wherein the assertive step-size amplifies the magnitude of perturbations between successive iterates. In this transient regime, the third-order tensor correction term $y_{kt}$, the regularization component $-\rho \frac{g_k^T d_{k-1}}{\delta _k} y_{kt}$, and the truncated conjugate coefficient $\beta _k^{\textrm{NE}+}$ have not yet accumulated sufficient historical information, resulting in oscillatory behavior. As the normalization factor $\delta _k$ and the corrected vector $y_{kt}$ progressively stabilize, the search direction autonomically gravitates toward a steady descent channel consonant with the sufficient descent property, whereupon the initial fluctuations are effectively suppressed. This dynamic trajectory serves to validate the intrinsic robustness of the three-term conjugate architecture and the tensor correction mechanism in accommodating a heterogeneous spectrum of step-size specifications.

On the nonconvex ERM problem, the convergence behavior of the TASCG algorithm on the Adult and Splice datasets is comparable to that of SGD when the same step size is employed. However, when the step size is increased to an appropriate range, the convergence speed of TASCG improves further. This result further indicates that a judiciously increased step size contributes to enhancing the overall optimization performance of the TASCG algorithm in nonconvex empirical risk minimization settings.

Next, we investigate the performance of the TASCG-VR algorithm. The experiment employs the same parameter configuration as that used for TASCG, with the step size of SVRG fixed at 0.025. The step sizes of TASCG-VR are set to 0.025, 0.03, and 0.035, respectively, in order to systematically analyze the impact of step size variation on convergence behavior.

A noteworthy phenomenon emerges upon examination of the results pertaining to the Adult dataset as depicted in Fig. 3: TASCG-VR exhibits a more precipitous initial descent relative to SVRG, yet subsequently encounters a transient plateau spanning approximately the first 40 iterations, after which it resumes an accelerated decline. Ultimately, TASCG-VR attains markedly superior final convergence precision and rate in comparison to SVRG. This distinctive behavioral pattern admits of elucidation through an analysis of the construction of the search direction $d_k$. During the incipient phase, the momentum term $-g_k + \beta _k^{\textrm{NE}+} d_{k-1}$ and the local second-order curvature information embedded within the third-order tensor correction term $y_{kt}$ propel the algorithm expeditiously into a comparatively flat neighborhood of a local minimum. At this stage, the gradient norm decreases, and the effects of both $\beta _k^{\textrm{NE}+}$ and the regularization term are correspondingly reduced, which results in a brief stagnation phase. Nevertheless, TASCG-VR persistently assimilates higher-order curvature information via the $y_{kt}$ vector to refine its characterization of the local geometric landscape. As this curvature intelligence progressively accumulates, the recalibration of $\beta _k^{\textrm{NE}+}$ endows the search direction with sufficient escape momentum to traverse saddle points or surmount shallow local extremal barriers. Once extricated from this region, the algorithm-under the aegis of a more precise third-order correction-enters a superior descent trajectory, whereupon its subsequent convergence performance conspicuously eclipses that of SVRG. The absence of this phenomenon on the remaining datasets intimates that the loss surface associated with the Adult dataset possesses a distinctive nonconvex architecture, and that TASCG-VR, by virtue of its three-term conjugate direction augmented with third-order tensor corrections, manifests a pronounced capacity for extricating itself from such localized entrapments. Across the other benchmark datasets, the overall optimization efficacy of TASCG-VR substantially surpasses that of SVRG, and this relative advantage becomes even more pronounced under more aggressive step-size configurations.

From the combined results shown in Figs. 3 and 4, the proposed TASCG-VR algorithm exhibits a clear advantage over SVRG in terms of the function value reduction rate, demonstrating that its three-term conjugate search direction with third-order tensor correction possesses strong competitiveness in machine learning optimization problems. Furthermore, appropriately increasing the step size within a certain range can further accelerate the convergence speed of TASCG-VR and achieve lower final function values. The above experimental results indicate that a reasonable selection of the step size parameter plays a crucial role in enhancing the convergence efficiency and overall performance of the TASCG-VR algorithm.

To comprehensively evaluate the proposed algorithm, we compared TASCG and its variance-reduced variant TASCG-VR against several representative baselines: classical stochastic gradient descent (SGD), variance-reduction methods (SVRG, SARAH, SAGA), and adaptive step-size methods (RMSprop, Adam). The initial step size was uniformly set to *0.0001* for all methods, and the remaining hyperparameters were configured following the original recommendations or determined via grid search to ensure a fair comparison. Figures 5 and 6 show that the loss curves of SGD, SVRG, SARAH, SAGA, and RMSprop largely overlap and exhibit the slowest decrease, while Adam performs moderately, with faster initial progress that later slows. For Model 1, TASCG descends more steeply than Adam, and TASCG-VR achieves the best performance. For Model 2, Adam initially converges fastest, but our algorithms gradually catch up, with TASCG-VR surpassing Adam after approximately 50 iterations. Specifically, SGD lacks both curvature information and variance reduction; SVRG, SARAH, and SAGA employ variance reduction but still rely on conventional gradient steps without leveraging higher-order curvature; RMSprop and Adam adapt the step size yet fail to capture higher-order curvature. In contrast, TASCG uses a three-term conjugate direction with a third-order tensor correction to exploit curvature for efficient descent, and TASCG-VR further integrates variance reduction, leading to fast initial convergence and higher final accuracy. Overall, these results demonstrate that our algorithms consistently deliver superior optimization performance and robustness across different models.

## Conclusion

This paper has addressed the fundamental challenges of nonconvex stochastic optimization by proposing a novel algorithmic framework that integrates third-order curvature approximation with a stochastic three-term conjugate gradient method. The principal contributions of this work are threefold. First, we introduce an augmented search direction incorporating third-order tensor information that notably satisfies both the sufficient descent property and boundedness conditions without auxiliary assumptions. Second, under standard smoothness and bounded gradient variance conditions, we establish rigorous global convergence guarantees for the proposed TASCG algorithm and derive a stochastic first-order oracle complexity bound of $\mathcal {O}(\epsilon ^{-2})$, which matches the optimal efficiency benchmarks of classical stochastic gradient methods. Third, we incorporate variance reduction techniques into the TASCG framework to obtain TASCG-VR, which improves gradient estimation accuracy and further enhances convergence behavior.

Extensive numerical experiments on nonconvex support vector machine and empirical risk minimization problems demonstrate the consistent superiority of both TASCG and TASCG-VR over their first-order counterparts, SGD and SVRG, respectively. This performance advantage is particularly pronounced under more aggressive step-size regimes. The initial oscillatory transients observed on small-sample datasets, along with the pronounced escape behavior exhibited on the Adult dataset, underscore the capacity of the third-order tensor correction to accurately capture complex local curvature landscapes and to facilitate escape from suboptimal basins of attraction.

In summary, the TASCG and TASCG-VR algorithms proposed herein provide a principled yet computationally efficient means of incorporating higher-order geometric information into the stochastic conjugate gradient paradigm. Both variants exhibit robust convergence properties, compelling empirical performance, and broad applicability to large-scale nonconvex machine learning tasks. Several promising directions for future research emerge from this work. One natural extension involves adapting the third-order curvature approximation framework to accommodate more general classes of nonconvex regularizers and nonsmooth objective functions. Other researches could integrate the third-order tensor curvature approximation for fixed-income portfolios with DSGE and CGE frameworks to build a unified macro-financial analysis system. For instance, incorporating third-order curvature enhanced bond allocation strategies into a monetary DSGE model will improve the identification of interactions between yield curve dynamics and interest rate pass through. In addition, combining the optimized fixed-income allocation structure with a financial CGE model can simulate the impact of treasury bonds and interest rate bond portfolio adjustments on projects like government financing costs, infrastructure investment, and cross-sector capital flows. This combined approach would bridge micro-level high-order fixed-income optimization and macro general equilibrium analysis, providing a more comprehensive toolbox for evaluating bond market performances and macroeconomic regulations.

## Data Availability

The datasets generated and/or analysed during the current study are available in the UC Irvine Machine Learning Repository. The datasets used in the paper are: (Adult:https://doi.org/10.24432/C5XW20);(Covtype: https://doi.org/10.24432/C50K5N);(Sonar: https://doi.org/10.24432/C5T01Q);(Splice:https://doi.org/10.24432/C5M888).
